# The mitochondrial genome of *Mylabris mongolica* Dokhtouroff, 1887 (Coleoptera: Meloidae)

**DOI:** 10.1080/23802359.2022.2080601

**Published:** 2022-06-14

**Authors:** Xiaoxiao Song, Shujing Gao, Saqiri, Ning Wang, Shang Gao

**Affiliations:** a Institute of Grassland Research, Chinese Academy of Agricultural Sciences, Hohhot, China; b Grassland Station of Xilinhot Forestry and Grassland Bureau, Xilinhot, China; c Department of Entomology, College of Plant Protection, China Agricultural University, Beijing, China

**Keywords:** Mitochondrial genome, Meloidae, *Mylabris mongolica*, phylogeny

## Abstract

*Mylabris mongolica* Dokhtouroff, 1887 is a traditional medicine material and an important predator in China. The mitochondrial genome of *M. mongolica* is presented for the first time in this study. The mitogenome is 15,034 bp in length and comprises 13 protein-coding genes, 2 rRNA genes, 22 tRNA genes, and partial control region. The nucleotide composition of *M. mongolica* was 36.7% of A, 18.1% of C, 11.1% of G, and 34.1% of T. The phylogenetic results divide all Meloidae species into two clades. The genus *Mylabris* was retrieved as a paraphyletic group, with *Mylabris* having a closer relationship with *Hycleus* than other genera within Meloidae. This study provides useful genetic data for future studies on the phylogeny and evolution of Meloidae species.

## Introduction

*Mylabris mongolica* Dokhtouroff, 1887 are beetles of the family Meloidae, also known as blister beetles. It is a traditional Chinese medicine material and a common predator of grasshopper, widely distributed in China and Mongolia (Pan and Bologna 2014). In recent years, molecular markers were widely used in Meloidae phylogenetic studies (Liu et al. 2020). However, there have been few molecular analyses including *M. mongolica*, many studies have mainly focused on morphological research (Yang and Ren 2007; Ren and Niu 2011), biological characteristics (Dong et al. 2005), and medicine utilization (Tan et al. 1995; Fang et al. 2001). In the present study, we present the mitochondrial genome of *M. mongolica* and its evolutionary relationships based on all available Meloidae mitogenomes.

Adult specimens of *M. mongolica* were collected from Sunite Right Banner, Inner Mongolia, China (42°47'0.19″N, 112°40'21.83"E) on 16th June 2020 by Ning Wang and identified by Pan Zhao. The sample collection was permitted by the Xilinhot Forestry and Grassland Bureau. All animal operations and experimental procedures were approved by the Administration Committee of Experimental Animals of Inner Mongolia Province and the Ethics Committee of the Chinese Academy of Agricultural Sciences. The voucher specimen was deposited in the Entomological Museum of the Institute of Grassland Research, Chinese Academy of Agricultural Sciences (No. IGR600075, Ning Wang: wangningis@163.com). Genomic DNA was extracted from the whole body of a single adult using the TrueLib DNA Library Rapid Prep Kit (Nanjing, China). The DNA sample was sent to Beijing Biomarker Biotechnology Co., LTD for library construction, and sequenced on the Illumina Novaseq 6000 platform. The final filtered reads (6 GB) were assembled with IDBA-UD (Peng et al. 2012). The initial annotation was conducted by MITOS webserver (Bernt et al. 2013) for determining the location and direction of genes. The annotation was manually validated by multiple sequences alignment based on other published Meloidae mitogenomes in MEGA v7.0 (Kumar et al. 2016).

The mitogenome of *M. mongolica* is 15,034 bp in length (GenBank accession number: OK638152) including 13 PCGs, 22 tRNA genes, two rRNA genes, and partial control region. The mitogenome of *M. mongolica* is similar to the related species reported before (Du et al. 2016; Wu et al. 2018; Han et al. 2020; Jiang et al. 2020). The nucleotide composition shows a high AT bias, with 70.8% of A + T content (A = 36.7%, T = 34.1%, C = 18.1%, G = 11.1%). The A + T content of PCGs, tRNAs, and rRNAs are 69.3%, 74.5%, and 73.6% respectively. Six PCGs (*NAD2, COI, ATP8, NAD3, NAD5*, and *NAD6*) initiate with ATT codons, seven PCGs (*COII*, *COIII*, *ATP6*, *NAD4, NAD4L, CYTB,* and *NAD1*) initiate with ATG codons. The typical termination codon TAA is assigned to 12 PCGs, only *NAD1* terminates with TAG.

**Figure 1.**
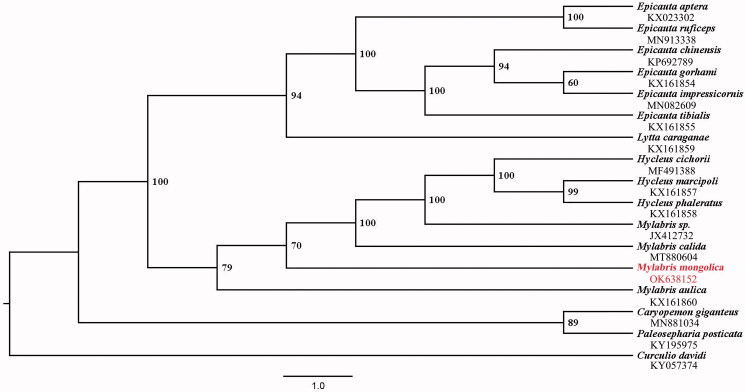
Maximum-likelihood (ML) phylogenetic tree of 17 Coleoptera species. The ML bootstrap is labeled at each node. The newly sequenced mitochondrial genome was highlighted in red.

Phylogenetic analysis was performed based on the nucleotide sequences of the mitochondrial genome of 14 species in four subfamilies from Meloidae, two species from Chrysomelidae, and one species (outgroup) from Curculionidae. The phylogenetic tree was constructed by IQ-TREE v2.0.6 (Bui et al. 2020) in PhyloSuite v1.2.2 (Zhang et al. 2020) based on 13 concatenated PCGs by using the maximum likelihood (ML) method. The results showed that *M. mongolica* along with the other three *Mylabris* species and three *Hycleus* species clustered together, but with the genus *Mylabris* being paraphyletic (Figure 1). The close relationship between *Mylabris* and *Hycleus* revealed in this study was consistent with a previous study (Jiang et al. 2020). The mitochondrial genome data of *M. mongolica* would be helpful for further phylogenetic and evolutionary analysis of Meloidae.

## Data Availability

The genome sequence data that support the findings of this study are openly available in GenBank of NCBI (https://www.ncbi.nlm.nih.gov/) under the accession No. OK638152. The associated BioProject, Sequence Read Archive (SRA), and Bio-sample accession numbers are PRJNA776618, SRR16768031, and SAMN22811307, respectively.
